# Analysis of polycomb repressive complex 2 (PRC2) subunits in *Picea abies* with a focus on embryo development

**DOI:** 10.1186/s12870-023-04359-9

**Published:** 2023-07-01

**Authors:** Tianqing Zhu, Jiwen Hu, Xiaowei Yang, Lisheng Kong, Juanjuan Ling, Junhui Wang, Sanping An

**Affiliations:** 1 State Key Laboratory of Tree Genetics and Breeding, Chinese Academy of Forestry, Haidian District, Dongxiaofu 1, Beijing, 100091 People’s Republic of China; 2 State Key Laboratory of Tree Genetics and Breeding, Key Laboratory of Tree Breeding and Cultivation of State Forestry and Grassland Administration, Research Institute of Forestry, Chinese Academy of Forestry, Beijing, 100091 PR China; 3 Department of Biology, Centre for Forest Biology, University of Victoria, Victoria, BC V8P 5C2 Canada; 4 Xiaolongshan Forestry Protection Center of Gansu Province, Tianshui, 741020 Gansu PR China

**Keywords:** H3K27me3, Polycomb group, Norway spruce, Seed development, Somatic embryogenesis

## Abstract

**Background:**

Conserved polycomb repressive complex 2 (PRC2) mediates H3K27me3 to direct transcriptional repression and has a key role in cell fate determination and cell differentiation in both animals and plants. PRC2 subunits have undergone independent multiplication and functional divergence in higher plants. However, relevant information is still absent in gymnosperms.

**Results:**

To launch gymnosperm PRC2 research, we identified and cloned the PRC2 core component genes in the conifer model species *Picea abies*, including one *Esc/FIE* homolog *PaFIE*, two *p55/MSI* homologs *PaMSI1a* and P*aMSI1b*, two *E(z)* homologs *PaKMT6A2* and *PaKMT6A4*, a *Su(z)12* homolog *PaEMF2* and a *PaEMF2-like* fragment. Phylogenetic and protein domain analyses were conducted. The *Esc/FIE* homologs were highly conserved in the land plant, except the monocots. The other gymnospermous PRC2 subunits underwent independent evolution with angiospermous species to different extents. The relative transcript levels of these genes were measured in endosperm and zygotic and somatic embryos at different developmental stages. The obtained results proposed the involvement of *PaMSI1b* and *PaKMT6A4* in embryogenesis and *PaKMT6A2* and *PaEMF2* in the transition from embryos to seedlings. The *PaEMF2-like* fragment was predominantly expressed in the endosperm but not in the embryo. In addition, immunohistochemistry assay showed that H3K27me3 deposits were generally enriched at meristem regions during seed development in *P. abie*s.

**Conclusions:**

This study reports the first characterization of the PRC2 core component genes in the coniferous species *P. abies*. Our work may enable a deeper understanding of the cell reprogramming process during seed and embryo development and may guide further research on embryonic potential and development in conifers.

**Supplementary Information:**

The online version contains supplementary material available at 10.1186/s12870-023-04359-9.

## Introduction

Conserved polycomb repressive complex 2 (PRC2) mediates tri-methylation of histone H3 lysine 27 (H3K27me3) to direct widespread transcriptional repression and control cell proliferation, cell fate determination, and cell differentiation at various developmental stages in both animals and plants [1]. The basic plant body has established during embryogenesis [2]. Mutations affecting PRC2 subunits lead to embryo lethality in both plants and animals [3, 4]. Plants possess several different PRC2 complexes to direct cell differentiation at various developmental stages [1–8]. Gymnosperms and angiosperms constitute the seed plants. The plant PRC2 subunits have been primarily studied in angiosperms. However, little is known about those of gymnosperms.

The PRC2 complex was first identified in *Drosophila melanogaster* and contains four core components: enhancer of zeste (E(z)), a histone methyltransferase; suppressor of zeste 12 (Su(z)12), a zinc finger protein; extra sex combs (Esc), a WD40 domain protein; and p55, also a Trp-Asp (WD) repeat protein [9]. The evolutionarily conserved SET domain of E(z) constitutes the catalytic site of lysine methyltransferases (KMTs) [10]. Su(z)12 has a VRN2-EMF2-FIS2-Su(z)12 (VEFS)-box at the C-terminal region, which is usually associated with a zinc-finger domain [11, 12]. The components of PRC2 have duplicated and diversified their function during green lineage evolution. Arabidopsis has 12 homologs of Drosophila PRC2 subunits: three E(z) homologs CLF, SWINGER (SWN) and MEDEA (MEA) [13–15]; three Su(z)12 homologs EMBRYONIC FLOWER 2 **(**EMF2), VERNALIZATION2 (VRN2) and FERTILISATION INDEPENDENT SEED2 (FIS2) [11, 16, 17]; one Esc homolog FERTILIZATION INDEPENDENT ENDOSPERM (FIE) [18]; and five p55 homologs MULTICOPY SUPPRESSOR OF IRA (MSI) 1–5 [19].

FIE and MSI1 are common components of all three variants of the Arabidopsis PRC2 complex. Most green lineage species have a single copy of FIE, whereas there was an expansion in the monocot crops [20]. CLF or SWN associate with EMF2 to form the variant EMF-PRC2 or associate with VRN2 to form the variant VRN-PRC2. They have partially redundant functions [15]. MEA orthologs only specifically arose in Cruciferae [21]. It was proposed that the CLF and MEA/SWN lineages separated before the divergence of monocots and dicots, whereas a more recent duplication produced the separation between MEA and SWN [22]. MEA associates with FIS2 to form the variant FIS-PRC2 [6, 7]. EMF2 is the most ancient Su(z)12. VRN2 and FIS2 orthologues have only been identified in dicots [20, 21, 23].

*FIE* gets its name because of its function in suppressing endosperm development until fertilization occurs [18]. Mutations in FIE and MSI1 allow endosperm development without fertilization [18, 24, 25]. In Arabidopsis, FIS-PRC2 regulates gametophyte and endosperm development. Maternal allele mutants affecting any components of FIS-PRC2 failed in endosperm cellularization and embryogenesis in Arabidopsis [14, 17, 18, 24]. Simonini et al. (2021) demonstrated that the requirement of FIS-PRC2 in endosperm and embryonic development is autonomous. In contrast, single or double mutants of CLF, SWN, and EMF2/VRN2 develop normal seeds [15]. The EMF- and VRN- PRC2s regulate sporophyte development [1–3] and are required for breaking seed dormancy [4, 5]. In rice (*Oryza sativa*), mutation of *OsEMF2a* induces autonomous endosperm development [2], while *OsEMF2b* controls seed dormancy and seedling growth [26]. In addition, PRC2 regulates genomic imprinting, especially expression of maternally expressed genes. The imprint seems to be erased during late embryo or early seedling development [20, 27].

The patterning and morphogenesis of seed development are different between gymnosperms and angiosperms. In angiosperms, a diploid zygote and a triploid central cell are formed after double fertilization. The zygote develops into an embryo through regular cell divisions, while the central cell develops into endosperm that is destined to support zygotic embryo (ZE) development. Without such a double fertilization mechanism, the haploid female gametophyte tissue formed by the development of unfertilized macrospores serves as the endosperm in gymnosperms. The zygotes of coniferous species undergo several rounds of nuclear duplication without cytokinesis, and there is no clear asymmetric cell division [28]. The process of somatic embryogenesis, which morphologically resembles its zygotic counterparts, has been proven to be a valuable tool for studying embryo development in conifers. The callus proliferates in the presence of auxin and cytokinin. Withdrawal of auxin and cytokinin induces early somatic embryos (SE) from embryonic callus (EC). The early SEs further develop into l mature SEs in the presence of abscisic acid. Partial desiccation treatment is needed for full maturation of SEs [29]. Nakamura et al. (2020) reported a significant role of H3K27me3 in the embryonic development of Norway spruce (*Picea abies*). According to their study, the H3K27me3 level was much lower in the EC than in the nonembryonic callus (NEC) but markedly increased upon SE induction. Many embryogenesis-related homologs, such as *ABI3*, *AIL5*, *FUS3*, *ESR2/DRNLs*, *CUC1* and *CUC2*, showed differential H3K27me3 enrichment between EC and NEC. Approximately 40% of *P. abies* genes that had Arabidopsis H3K27me3-marked homology were also H3K27me3-marked, which suggests conserved epigenetic mechanisms in the regulation of embryogenesis between Arabidopsis and *P. abies* [30].

In this study, we identified and cloned the PRC2 core component genes in *P. abies*, including one *Esc/FIE* homolog *PaFIE*, two *p55/MSI* homologs *PaMSI1a* and *PaMSI1b*, two *E(z)* homologs *PaKMT6A2* and *PaKMT6A4*, a *Su(z)12* homolog *PaEMF2* and a *PaEMF2-like* fragment. Phylogenetic analysis was performed, and the transcript levels of these *P. abies* PRC2 genes were analyzed in endosperm and ZEs and SE at different developmental stages. In addition, we discussed the role of PRC2-directed H3K27me3 in cell reprogramming. Our work provides novel knowledge about PRC2 subunits in *P. abies* and can guide further studies on the molecular mechanism of histone modification during embryogenesis and development of coniferous species.

## Results

### Cloning and phylogeny of *P. abies PRC2* genes

The reproductive development process of conifers is very different from that of angiosperms, yet there is currently a lack of relevant research on the PRC2 complex in conifers. In order to carry out related studies on the PRC2 complex of conifers, we cloned PRC2 genes and conducted the phylogenetic analysis. We conducted a BLAST search on the homologs of Arabidopsis PRC2 genes in the *P. abies* genome database [31]. The resulting sequences were clearly incomplete (Table S1). The genes are highly conserved among coniferous species. We further searched for PRC2 homologs in all *Picea* species. The CDSs of one *P. abies Esc/FIE* and two *P. abies p55/MSI* (*PaMSI1a* and *PaMSI1b*) homologs were cloned with reference to *P. sitchensis* homologous genes. *P. abies Su(z)12* (*PaEMF2*) and *P. abies E(z)* (*PaKMT6A2* and *PaKMT6A4*) homologs were isolated using degenerate primers targeting the homeodomain sequences from *AtEMF2* and *CLF* respectively. The isolated homeodomain sequences were extended by 3’ and 5’ RACE. Another 651 bp *P. abies* EMF2-like fragment was isolated (Supplementary File 1). The obtained CDSs were translated for conserved domain analysis.

**Fig. 1:**
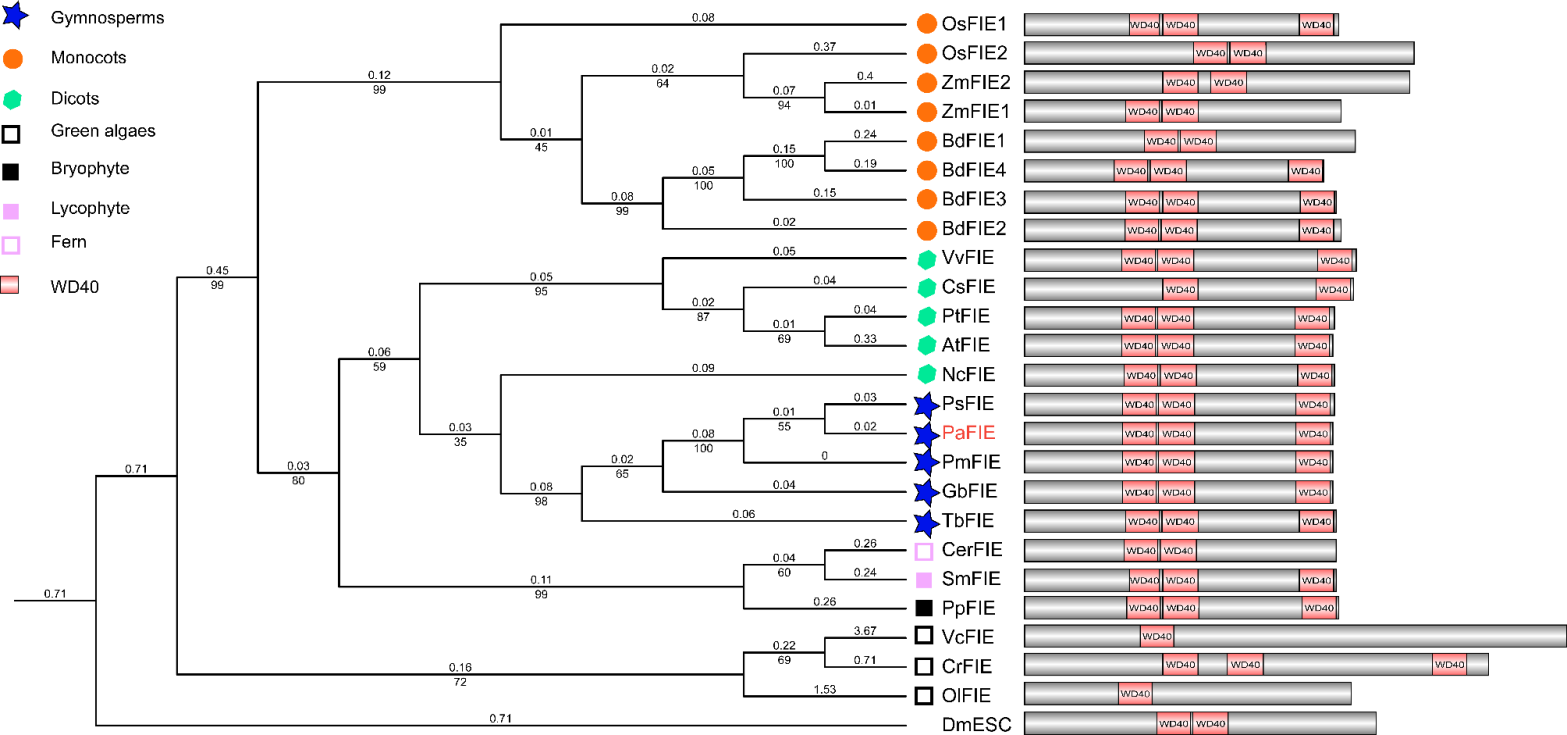
Phylogenetic tree of Esc/FIE homologs. The tree was made using maximum likelihood (ML) and amino acid data. Branch lengths are indicated by the number on the respective branch. Bootstrap support values are indicated by the number beneath the respective branch. Esc/FIE is highly conserved between gymnosperm and dicots. The tree contains sequences from green algae (*Ostreococcus lucimarinus, Ol*; *Volvox carteri, Vc*; and *Chlamydomonas reinhardtii*, *Cr*. ), bryophytes (*Physcomitrella patens*, *Pp*), lycophytes (*Selaginella moellendorffii*, *Sm*), ferns (*Ceratopteris richardii*, *Cer*), gymnosperms (*P. abies*, *Pa*., *Pseudotsuga menziesii, Pm*, *Taxus baccata*, *Tb*, *Gnetum montanum* Gm, *Pinus sylvestris, Ps* and *Ginkgo biloba*, *Gb*), and angiosperms (dicotyledons: *A. thaliana*, *At*, *Nymphaea colorata*, *Nc, Citrus sinensis*, *Cs*, *Populus trichocarpa*. *Pt* and *Vitis vinifera*, *Vv*; monocots: *Zea mays*, *Zm*, *Oryza sativa*, *Os, Brachypodium distachyon*, *Bd.*). *Drosophila melanogaster* (*Dm*) was used as an outgroup. The P. *abies* sequences are indicated by red bold font. Most Esc/FIE proteins contain two or three WD40 domains

Esc/FIE is one of the most conserved PRC2 proteins. PaFIE was 70% identical to Arabidopsis FIE with 99% coverage. All gymnospermous species Esc/FIE homologs examined here had three WD40 domains, two closely linked and one at the C-terminus. The WD40 repeats are necessary to bind directly to E(z). Most of the species had only one Esc/FIE homolog gene, except the monocots (Fig. 1 and Supplementary File 2). Several studies have confirmed subfunctionalization of Esc/FIE homologs in monocots. For example, *OsFIE1* functions predominantly in late seed development [32], and *OsFIE2* is vital for rice reproduction and endosperm formation [33]. These results suggested an independent evolution of Esc/FIE genes in monocots after the divergence of dicots and monocots.

**Fig. 2:**
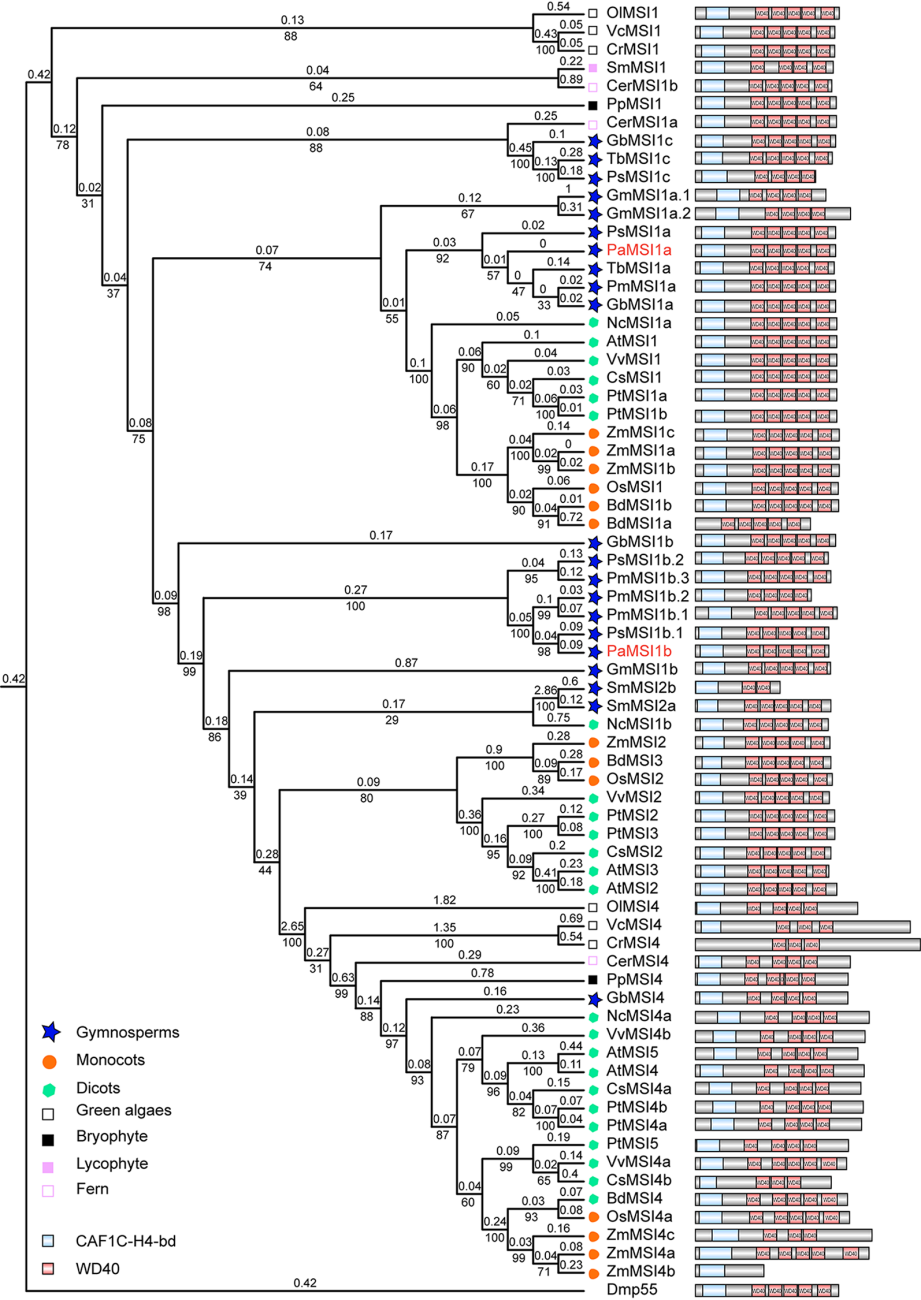
Phylogenetic tree of p55/MSI homologs. The tree was made using maximum likelihood (ML) and amino acid data. Branch lengths are indicated by the number on the respective branch. Bootstrap support values are indicated by the number beneath the respective branch. The MSI1 homolog has been duplicated in gymnosperm. The tree contains sequences from green algae (*Ostreococcus lucimarinus, Ol*; *Volvox carteri, Vc*; and *Chlamydomonas reinhardtii*, *Cr*. ), bryophytes (*Physcomitrella patens*, *Pp*), lycophytes (*Selaginella moellendorffii*, *Sm*), ferns (*Ceratopteris richardii*, *Cer*), gymnosperms (*P. abies*, *Pa*., *Pseudotsuga menziesii, Pm*, *Taxus baccata*, *Tb*, *Gnetum montanum* Gm, *Pinus sylvestris, Ps* and *Ginkgo biloba*, *Gb*), and angiosperms (dicotyledons: *A. thaliana*, *At*, *Nymphaea colorata*, *Nc, Citrus sinensis*, *Cs*, *Populus trichocarpa*. *Pt* and *Vitis vinifera*, *Vv*; monocots: *Zea mays*, *Zm*, *Oryza sativa*, *Os, Brachypodium distachyon*, *Bd.*). *Drosophila melanogaster* (*Dm*) was used as an outgroup. The P. *abies* sequences are indicated by red bold font. Most of the p55/MSI proteins contain subunit C of the CAF1 complex (CAF1C-H4-bd) and three or five WD40 domains

p55/MSI members are also WD40 repeat-containing proteins (Fig. 2). We cloned two *p55/MSI* homologs in *P. abies* (Supplementary File 1). The *p55/MSI* genes can be divided into three clades, i.e., MSI1, MSI2/3 and MSI4/5, which are primarily based on the angiosperm species [21]. The MSI1 clade contains genes from all early diverging plants (algaes, bryophytes, lytophytes and ferns). Three gymnospermous genes from *T. baccata*, *P. sylvestris* and *G. biloba* were more related with these ancient MSI1 homologs. All gymnospermous species examined here had at least two MSI1 homologs. One copy, named as MSI1a, was clustered with the angiosperm MSI1 clade. While the other, named as MSI1b, were relatively distant from the angiosperm MSI1. We only identified one gymnospermous gene that was clustered with the MSI4/5 clade, which contains genes from all early diverging plants except S. *moellendorffii*. Of note, the current gymnospermous genomes are difficult to assemble exactly due to their large size and repeat-rich sequences [31, 34, 35]. At this point, we cannot exclude the existence of other p55/MSI members in *P. abies*.

**Fig. 3:**
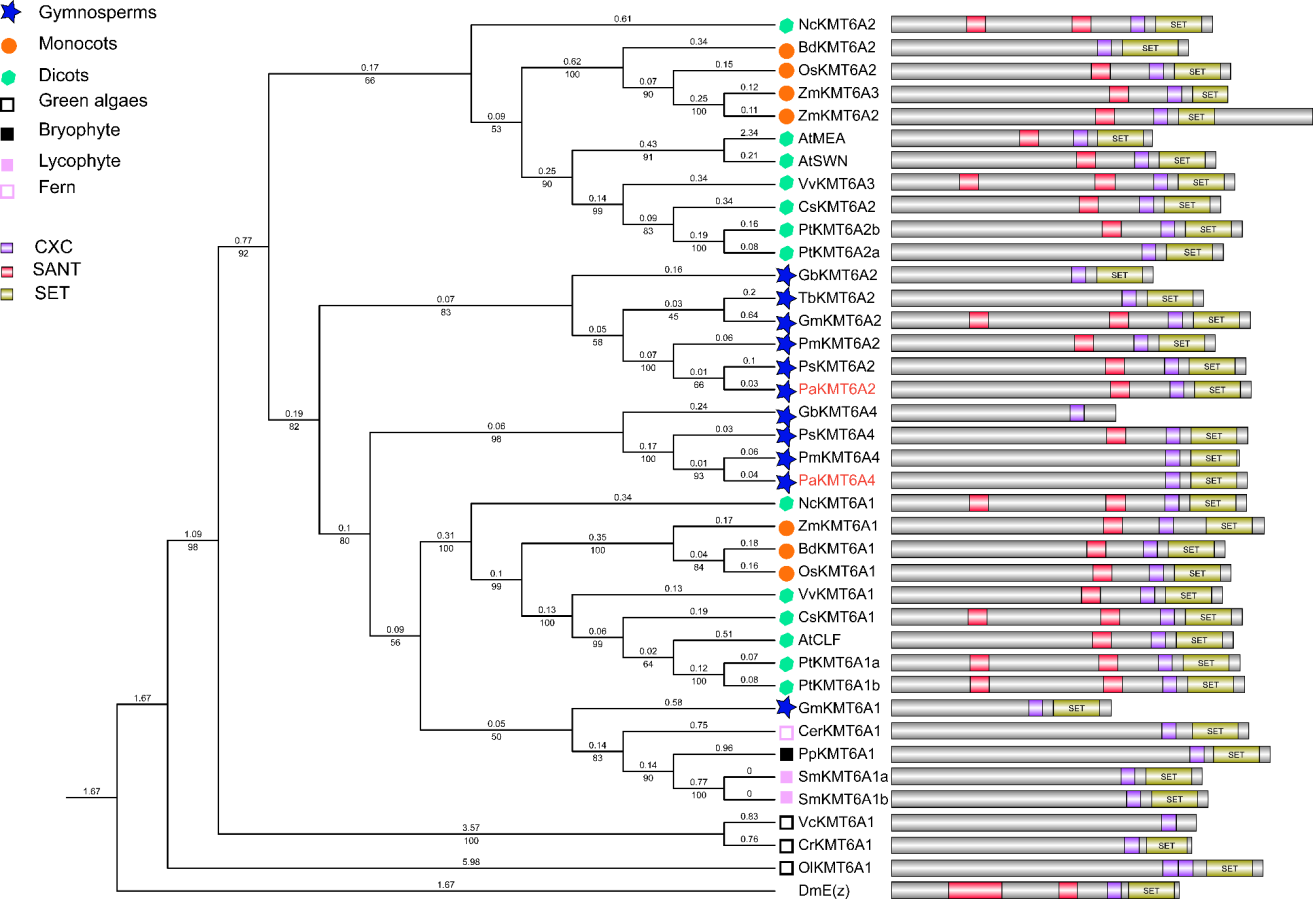
Phylogenetic tree of E(z) homologs. The tree is made using maximum likelihood (ML) and amino acid data. Branch lengths are indicated by the number on the respective branch. Bootstrap support values are indicated by the number beneath the respective branch. The phylogenetic analyses showed that E(z) homologs underwent independent radiation after the split between angiosperms and gymnosperms. The tree contains sequences from green algae (*Ostreococcus lucimarinus, Ol*; *Volvox carteri, Vc*; and *Chlamydomonas reinhardtii*, *Cr*. ), bryophytes (*Physcomitrella patens*, *Pp*), lycophytes (*Selaginella moellendorffii*, *Sm*), ferns (*Ceratopteris richardii*, *Cer*), gymnosperms (*P. abies*, *Pa*., *Pseudotsuga menziesii, Pm*, *Taxus baccata*, *Tb*, *Gnetum montanum* Gm, *Pinus sylvestris, Ps* and *Ginkgo biloba*, *Gb*), and angiosperms (dicotyledons: *A. thaliana*, *At*, *Nymphaea colorata*, *Nc, Citrus sinensis*, *Cs*, *Populus trichocarpa*. *Pt* and *Vitis vinifera*, *Vv*; monocots: *Zea mays*, *Zm*, *Oryza sativa*, *Os, Brachypodium distachyon*, *Bd.*). *Drosophila melanogaster* (*Dm*) was used as an outgroup. The *P. abies* sequences are indicated by red bold font. Most E(z) proteins have SET, SANT and CXC domains

Two SET domain-containing E(z) homologs in *P. abies*, *PaKMT6A2* and *PaKMT6A4*, were cloned in our study. Most of the gymnospermous species examined here had two H3K27 methyltransferase genes (Supplementary File 2) after excluding the putative genes that lack the SET domain. Our data suggest that E(z) homologs underwent independent radiation after the split between angiosperms and gymnosperms (Fig. 3). This agreed with previous publication [30]. Of note, AtMEA was clustered with AtSWN in our study (Fig. 3). This might be caused by long branch attraction [36]. The SWN proteins only exist in dicots and monocots, and the MEA proteins have only been found in the Cruciferae [21]. It should be better to include more Cruciferae species to analyze the phylogenetic relationship between MEA and SWN.

**Fig. 4:**
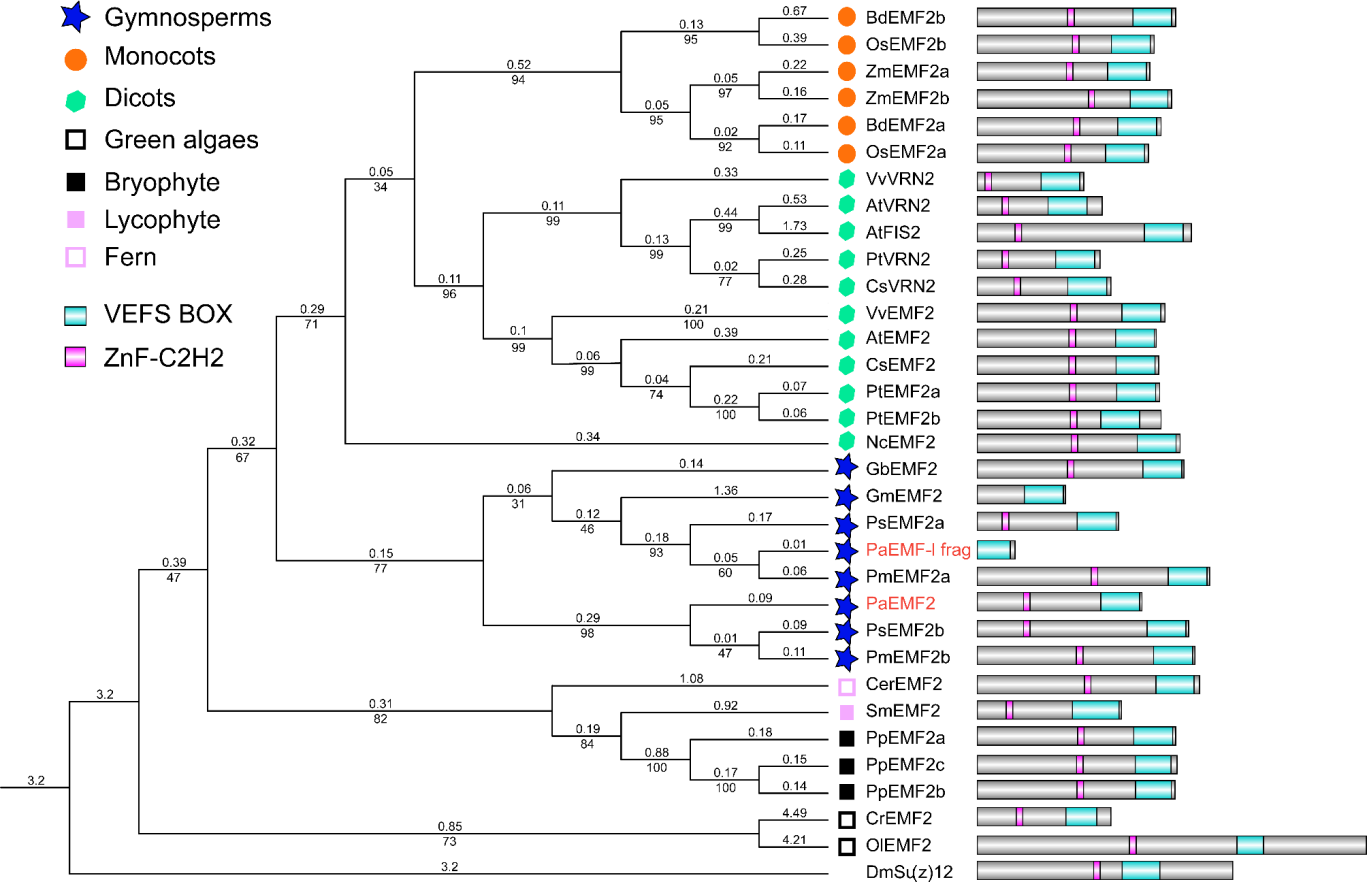
Phylogenetic tree of Su(z)12 homologs. The tree is made using maximum likelihood (ML) and amino acid data. Branch lengths are indicated by the number on the respective branch. Bootstrap support values are indicated by the number beneath the respective branch. The phylogeny of *Su(z)12* homologs was similar to that of known plant evolution. The tree contains sequences from green algae (*Ostreococcus lucimarinus, Ol*; *Volvox carteri, Vc*; and *Chlamydomonas reinhardtii*, *Cr*.), bryophytes (*Physcomitrella patens*, *Pp*), lycophytes (*Selaginella moellendorffii*, *Sm*), ferns (*Ceratopteris richardii*, *Cer*), gymnosperms (*P. abies*, *Pa*., *Pseudotsuga menziesii, Pm*, *Gnetum montanum* Gm, *Pinus sylvestris, Ps* and *Ginkgo biloba*, *Gb*), and angiosperms (dicotyledons: *A. thaliana*, *At*, *Nymphaea colorata*, *Nc, Citrus sinensis*, *Cs*, *Populus trichocarpa*. *Pt* and *Vitis vinifera*, *Vv*; monocots: *Zea mays*, *Zm*, *Oryza sativa*, *Os, Brachypodium distachyon*, *Bd.*). *Drosophila melanogaster* (*Dm*) was used as an outgroup. The *P. abies* sequences are indicated by red bold font. All Su(z)12 proteins have a ZnF-C2H2 domain and the VEFS box

We identified and cloned two EMF2-like sequences in *P. abies* (PaEMF2 and PaEMF2-like fragment (Supplementary File 1). All Su(z)12 homologs have a VEFS box at the C-terminal region and a C2H2-type zinc finger (ZnF-C2H2) domain except GmEMF2 (312 AA.) and PaEMF2-like fragment (130 AA.), which were due to the incomplete sequences (Fig. 4). These two domains are conserved in both animals and plants. The phylogeny of *Su(z)12* homologs was similar to that of known plant evolution. The gymnosperm and angiosperm clades were separated. Most of the gymnospermous species examined here had two *Su(z)12* homologs (Supplementary File 2).

### Expression analysis of the *P. abies PRC2* genes

Transcript levels of the *P. abies PRC2* genes were analyzed in endosperm, zygotic and somatic embryos (ZEs and SEs) at different developmental stages. The embryogenesis process in conifers could be divided into three phases: i) Early embryogeny, all stages after elongation of the suspensor and before the establishment of the root meristem, ii) Late embryogeny, during which the meristems are established and iii) Maturing development of the embryo [28]. The ‘early’, ‘late’ and ‘mature’ embryos mentioned later can be referred to these three phases. Although they may be not very accurate. Samples collected at same time point, excluded the ones that were obviously morphologically different, were grouped together.

**Fig. 5:**
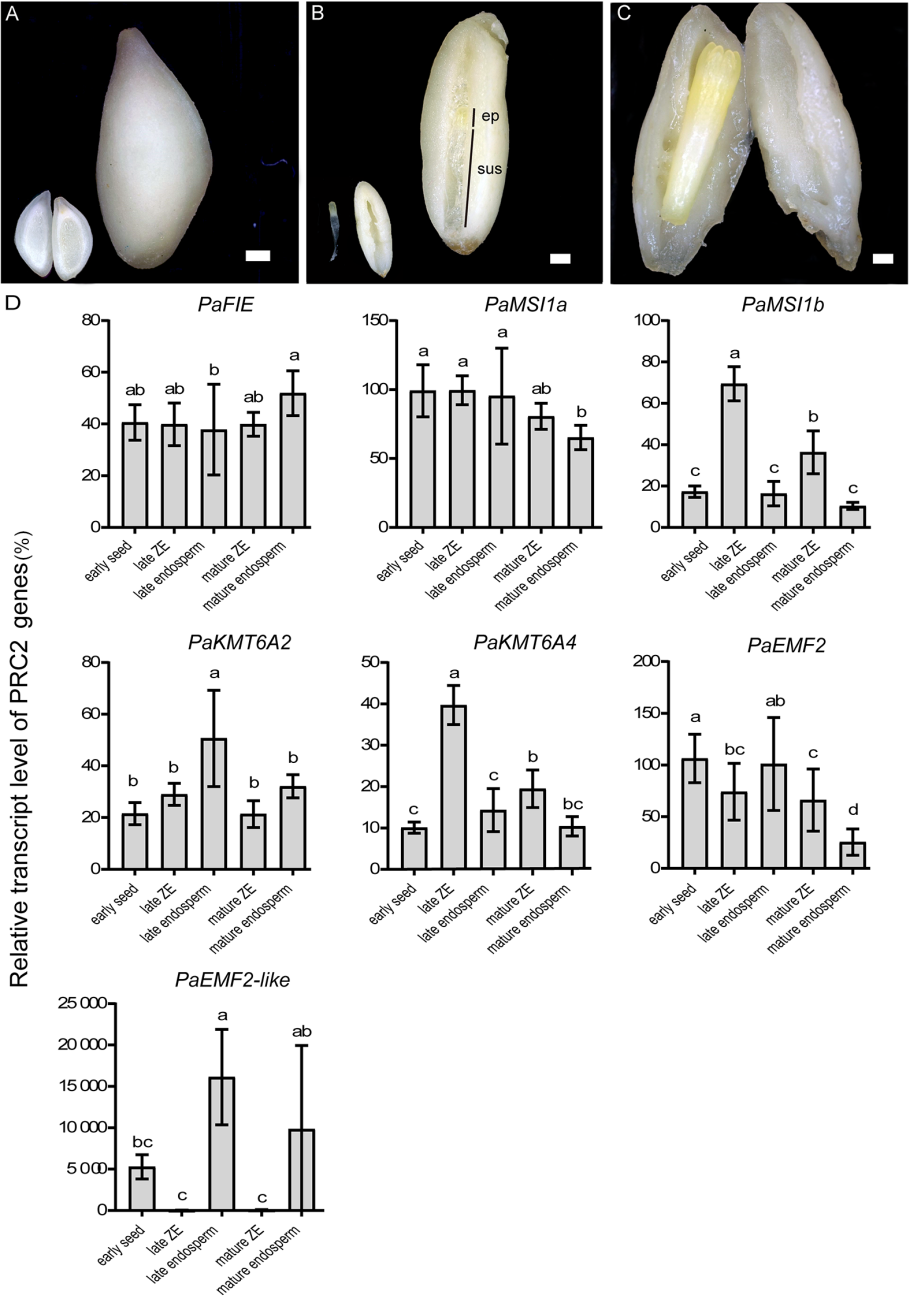
Expression analysis of the *P. abies PRC2* subunit genes in seeds. Bars = 250 μm. **(A)** Early seed, inset: longitudinal section of the early seed. **(B)** Longitudinal section of seed contains late zygotic embryo (ZE). ep, embryo proper; sus, suspensor. Inset: late ZE (left) and endosperm (right). **(C)** Seed contains mature ZE. **(D)** Relative transcript level of the *P. abies PRC2* genes (± SEM). Seedling needles were used as a somatic control. All data were relative to the transcript level of *PaMSI1a* in early seed. Error bars indicate standard deviations (n = 3) between biological replicates. Means that do not share a letter are significantly different according to Duncan test (*p*-value < 0.05). All of the PRC2 genes, except *PaFIE*, showed higher transcript abundance in the samples at late stage than it at mature stage. The transcript level of *PaFIE*, *PaMSI1a* and *PaKMT6A2* were relatively stable among the seed samples. The higher transcript level of *PaKMT6A2* in late endosperm might be due to mix of samples from earlier stage. The transcript level of *PaMSI1b* and *PaKMT6A4* were higher in ZEs than in endosperms. The *PaEMF2-like* fragment was dominantly detected in endosperms and early seed, which also contained endosperm

Early seed, late ZE, late endosperm, mature ZE and mature endosperm were collected for expression analysis (Fig. 5A-C). Seedling needles were used as a somatic control. The transcript levels of most *PRC2* genes were low in needle and were higher in the samples at late stage than it at mature stage (Fig. 5D). *PaFIE* was the only genes that was stably expressed among all the seed samples. The transcript levels of *PaMSI1a* were not significantly different between ZE and endosperm. The transcript level of *PaKMT6A2* was higher in late endosperm than in the other samples, which might due to mix of samples from earlier developmental stage. The transcript levels of *PaKMT6A4* and *PaMSI1b* were higher in ZEs than in early seed and endosperms. *PaEMF2* did not show a clear expression pattern in these samples. In comparison, the *PaEMF2-like* fragment was extremely abundant in early seed and endosperm but not in ZEs (Fig. 5D).

**Fig. 6:**
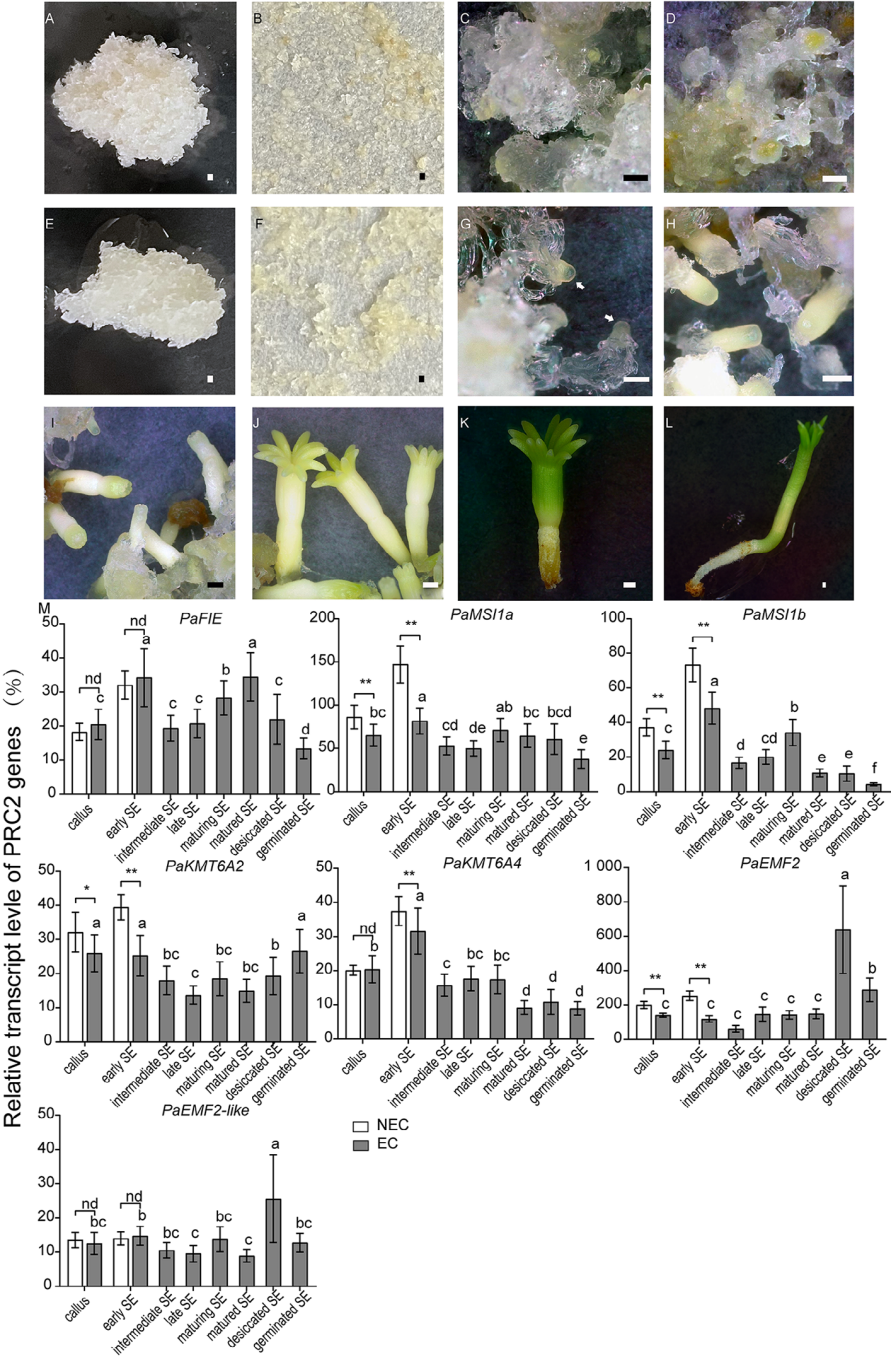
Expression analysis of the *P. abies PRC2* subunit genes during somatic embryo (SE) development and germination. Bar = 250 μm. **A-D**) Somatic embryogenesis of the nonembryonic callus (NEC), including **(A)** proliferating NEC; **(B)** cultures after one week on medium lacking auxin and cytokinin to stimulate differentiation of early SE; **(C)** cultures on maturation medium supplemented with ABA for one week; **(D)** cultures on maturation medium for three weeks. Of Note, no SE developed in NEC. **E-L**) Somatic embryogenesis and germination of embryonic callus (ECs), including **(E)** proliferating ECs; **(F)** cultures after one week on medium lacking auxin and cytokinin to stimulate differentiation of early SE; **(G)** cultures on maturation medium supplemented with ABA for one week, note the intermediate SE pointed by the white arrows; **(H)** cultures on maturation medium for 2 weeks, note the late SE; **(I)** maturing SE after 4 weeks on maturation medium; **(J)** Fully matured SE after 6 weeks on the maturation medium; **K**) desiccated SE after partial desiccation treatment; **L**) germinated SE after 4 weeks of germination. **M**) Relative transcript level of the *P. abies PRC2* genes (± SEM). All data were relative to the transcript level of *PaMSI1a* in the callus of NEC. Error bars indicate standard deviations (SD) (n = 3) between biological replicates. Means that do not share a letter are significantly different according to Duncan test (*p*-value < 0.05). Asterisks indicate the significant difference between NEC and EC samples according to least significance difference test (‘*’, *p*-value < 0.05; ‘**’, *p*-value < 0.01; and ‘nd’, no difference)

EC and NEC were used to study the expression of *P. abies* PRC2 genes at more embryo developmental stages, including the desiccation and germination stages. The somatic embryogenesis process was blocked in NEC. No SE could be observed in NEC after SE induction and maturation (Fig. 6A-D). In comparation, one week elimination of auxin and cytokinin induced early embryogeny in EC (Fig. 6E-F). The early SEs from EC developed into intermediate SEs (Fig. 6G), late SE (Fig. 6H), maturing SE (Fig. 6I) and maturated SE (Fig. 6J) on the maturation medium. After desiccation, the desiccated SE (Fig. 6K) germinated on the germination medium (Fig. 6K). The germinating SE (Fig. 6L) was defined by radicle elongation.

The transcript levels of the *P. abies* PRC2 genes were analyzed in these samples (Fig. 6M). Of note, both EC and NEC contain a mixed cell type and we cannot isolate individual SE until the mature phase. The transcript level of *PaMSI1a, PaMSI1b, PaKMT6A2* and *PaEMF2* were higher in NEC than in EC. These factors might lead to a higher level of H3K27me3 in NEC than in EC. After auxin and cytokinin elimination, one more PRC2 genes, *PaKMT6A4*, were higher expression in NEC than in EC. Four genes, *PaFIE, PaKMT6A4, PaMSI1a* and *PaMSI1b*, were upregulated in early SE. These genes might contribute to somatic embryogenesis. *PaKMT6A4* and *PaMSI1b* were lowly expressed after SE maturation. While the transcript level of *PaMSI1a* and *PaFIE* decreased gradually from matured SE to germinated SE. This might reflect a more specific expression pattern of these two gene with the growth of the plant. The transcript levels of *PaKMT6A2* and *PaEMF2* were relatively stable during embryogenesis and were significantly upregulated in desiccated SE. *PaKMT6A2*, *PaEMF2, PaFIE* and *PaMSI1a* might participate in the transition of embryo to seedling.

### Immunohistochemistry analysis of H3K27me3 in seeds at different developmental stages

**Fig. 7:**
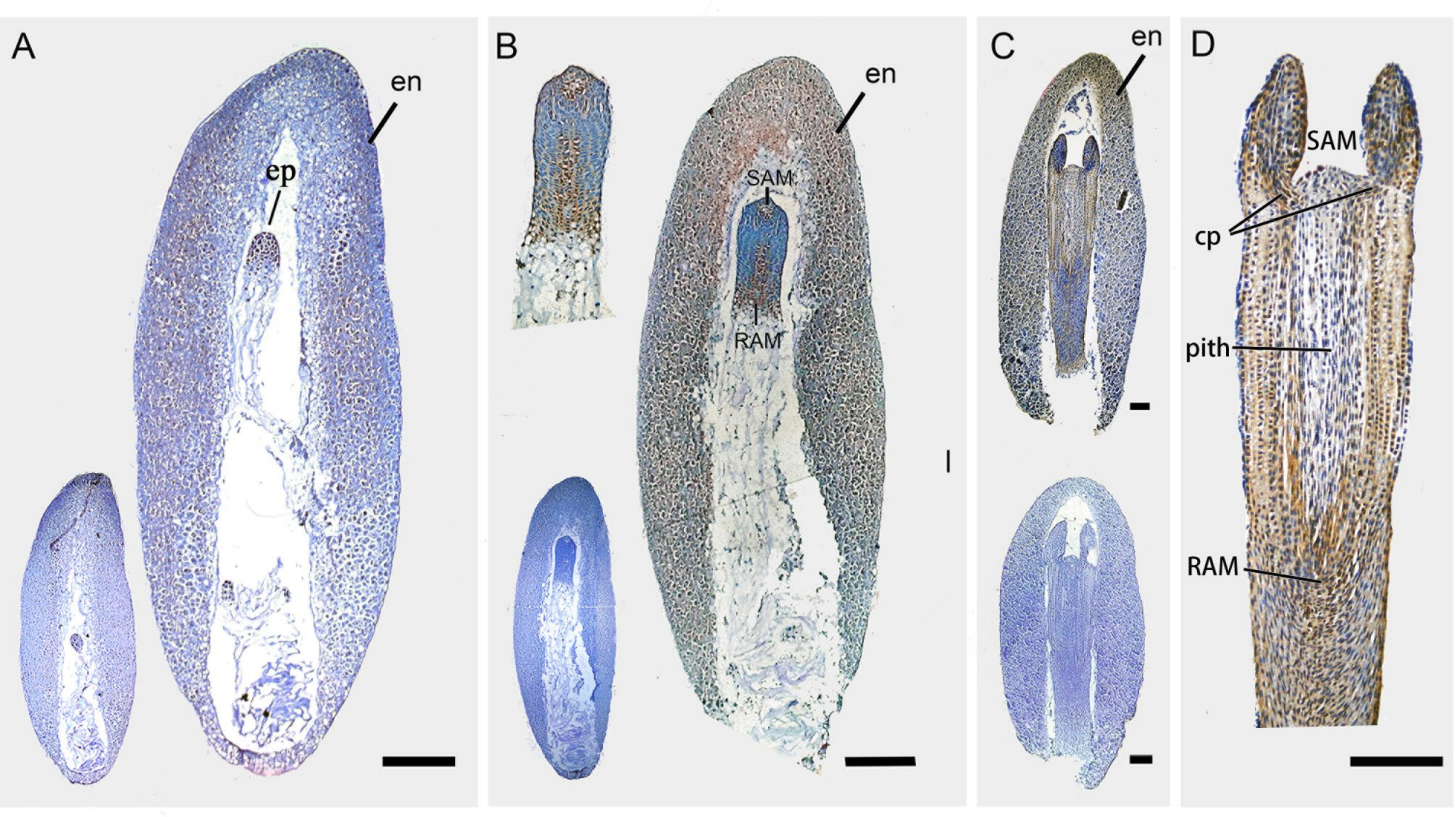
H3K27me3 immunolocalization in *Picea abies* seeds. Bars = 250 μm. **A-C**) H3K27me3 is widespread in the endosperm (en). **(A)** A seed contains an early zygotic embryo (ZE). Lower-left inset, control. Note that H3K27me3 is widespread in the embryonal proper (ep). **(B)** A seed contains a late ZE. Upper-left inset, enlarged late ZE with higher resolution; lower-left inset, control. Note that H3K27me3 is deposited at the shoot apical meristem (SAM) and root apical meristem (RAM) region. **(C)** A seed contains mature ZE (upper, anti-H3K27me3; lower, control). **(D)** Enlarged mature ZE with higher resolution. Note that H3K27me3 is particularly deposited at SAM, RAM, cytoledon primordia (cp.) and around the pith

To detect H3K27me3 deposition during seed development in *P. abies*, an H3K27me3 immunohistochemistry assay was performed. H3K27me3 was detected throughout the endosperm in all tested samples (Fig. 7A-C). Meanwhile, the embryo showed a specific H3K27me3 pattern during embryogenesis. In early ZE, H3K27me3 was detected in the whole embryo (Fig. 7A). Coniferous embryo lack restricted cell division during early embryogeny [28]. This prevents the formation of a distinct pattern in the early embryo. The universal H3K27me3 deposit might reflect extensive switch off genomic expression in the early embryo. In late ZE, H3K27me3 deposits were enriched at two regions along the embryo axis (Fig. 7B). One is at the apical of the embryonal proper. The other is from the center to the basal part of the embryonal proper. The H3K27me3 mark extended to the whole layer at the basal part of the embryonal proper (Fig. 7B). In conifer, the root and shoot apical meristem are established along the embryo axis during late embryogeny [28]. The root apical meristem forms near the center of the embryo. While the shoot apical meristem is at the distal part of the embryonal proper. The basal cells of the embryonal proper have been descripted as a distal stem cell type which give rise to suspensor during early and late embryogeny [37]. Procambium and cortex are also differentiated at this stage. In mature ZE, H3K27me3 deposits were all over the embryo with an enrichment around the pith, at the root apical meristem and the cotyledon primordia around distal end of the embryo (Fig. 7C, D). Apparently, the H3K27me3 deposit overlapped with the meristem region in the embryo of *P. abies*. In addition, more positive anti-H3K27me3 spots were detected in NEC than in EC in our study (Figure S1).

## Discussion

The PRC2 system arose even before the split of plants and animals [38]. It plays a key role in cell differentiation and pattern formation by mediating H3K27me3 regulation of gene expression [8, 39]. In this study, we identified the *P. abies* PRC2 genes, including all four types of PRC2 core components: one *Esc/FIE* homolog, *PaFIE*; two *p55/MSI1* homologs, *PaMSI1a* and *PaMSI1b*; two SET-domain-containing histone methyltransferase-encoding genes, *PaKMT6A2* and *PaKMT6A4*; a zinc finger protein-encoding gene, *PaEMF2*, and a *PaEMF2-like* fragment.

Our data showed that the *FIE* homologs were highly conserved in land plants, except in monocots, but the MSI1 homolog has been duplicated in gymnosperms (Figs. 1 and 2). *PaMSI1a* and *PaMSI1b* showed distinct transcript abundances in different tissues. During seed development, *PaMSI1b* was enriched in embryos (Fig. 5D). In contrast, the expression of *PaMSI1a* was stable among different tissues (Fig. 5D). During somatic embryogenesis, both *PaMSI1s* were up-regulate in respond to auxin and cytokinin elimination in EC (Fig. 6M). *MSI1* may have duplicated and diverged different functions in gymnosperms. The duplicated *PaMSI1b* might have a special involvement in coniferous embryogenesis. While the conserved *MSI1a* has a more general function in plant development. Additional studies will be required to further characterize and validate the involvement of *PaMSI1a* or *PaMSI1b* in the PRC2 system and subsequent H3K27me3 deposition.

In Arabidopsis, FIS-PRC2 regulates both embryo and endosperm development [8]. However, in our study, the *PaEMF2-like* fragment was particularly abundant in the endosperm-enriched samples (early seed and endosperms) (Fig. 5D). Endosperm and embryo patterning of *P. abies* are very different from those of angiosperms. *P. abies* endosperm originates from the female gametophyte and initiates development before fertilization. The H3K27me3 deposit in endosperm and embryo may be regulated by different PRC2 variants in *P. abies*.

**Fig. 8:**
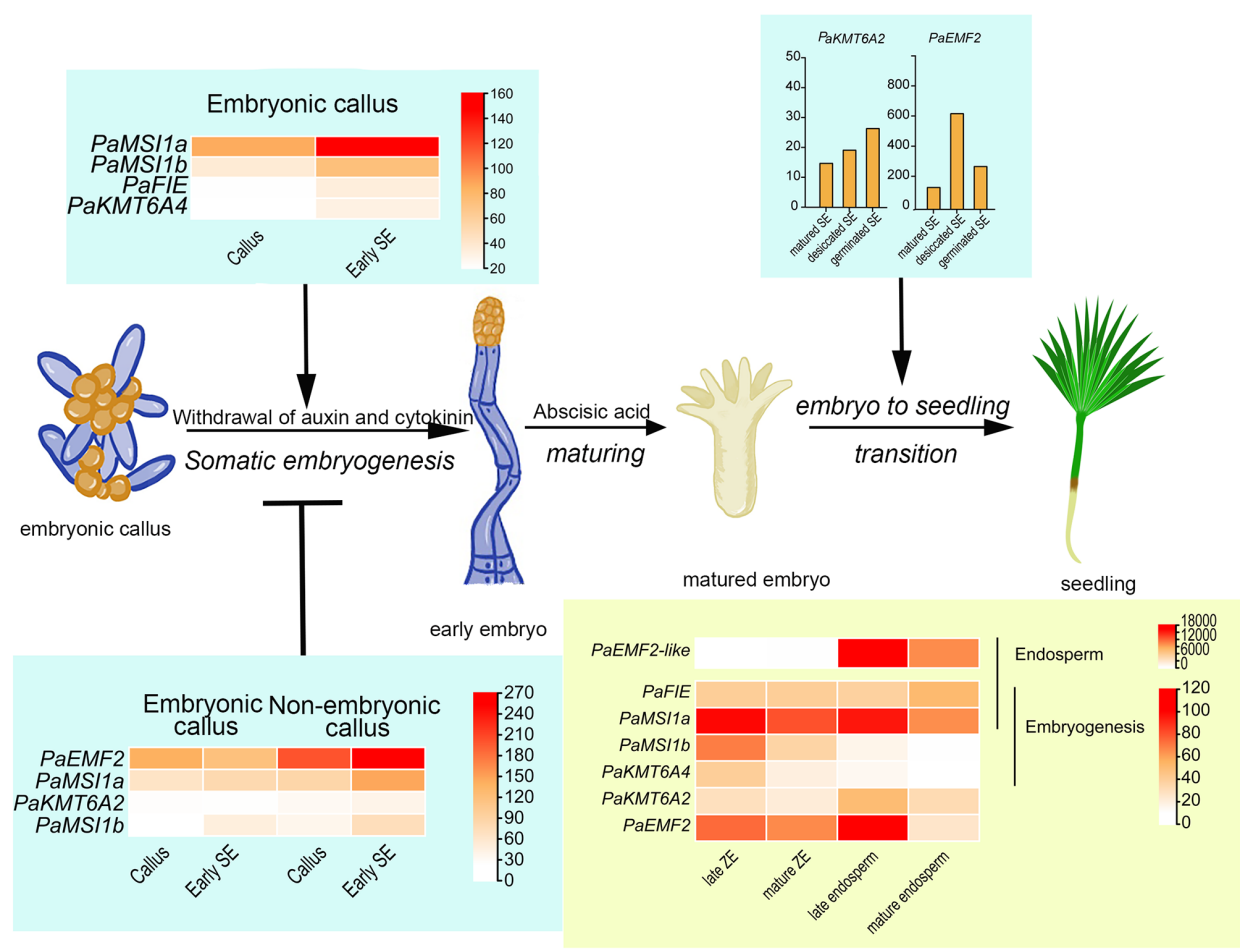
Sketch shows the coniferous embryogenesis and the developmental processes in which the PRC2 subunits participate in. In conifer, the fertilized zygotic nucleus divides into four free nuclei without cellularization. Then, after several round cell division, the basal tiers elongate to form a functional suspensor and the apical tiers form the embryonal proper. During somatic embryogenesis, the elimination of auxin and cytokinin stimulate the differentiation of early somatic embryos. Early embryogeny begins with the elongation of the embryonal suspensor. The zygotic and somatic embryogenesis are morphologically similar from early embryogeny phase. Embryo patterning, including the establishment of root apical meristem, shoot apical meristem, procambium, cotyledon primordia and cortex etc., are performed during early and late embryogeny. After maturation, embryos can germinate under suitable conditions. Our data suggested that *PaEMF2-like*, together with the generally expressed *PaMSI1a* and *PaFIE*, involve in the development of endosperm; no endosperm specific E(z) homolog was charactered in our study; *PaKMT6A4*, *PaMSI1s* and *PaFIE* were related with embryogenesis and might contribute to embryo patterning; no *Su(z)12* homolog was upregulated in early SE in our study; *PaKMTA2* and *PaEMF2*, together with *PaMSI1a* and *PaFIE*, might participate in the embryo to seedling transition. In addition, *PaKMT6A2, PaEMF2, PaMSI1a* and *PaMSI1b* might be antagonistic regulators of somatic embryogenesis

In the seeds, the transcript of *PaKMT6A4* was enriched in ZE (Fig. 5D), which suggested an role in embryo development. Furthermore, *PaKMT6A4*, together with *PaFIE* and *PaMSI1s*, were upregulated after SE induction (auxin and cytokinin elimination) (Fig. 6M). Consist with the previous study, in which the H3K27me3 level were increased after auxin and cytokinin elimination [30], these genes might be respond for the H3K27me3 deposit during embryo patterning. *PaEMF2-like* was also detected in the SE samples, but its abundance was much lower than *PaEMF2.* Since our calli were induced from peeled immature seed. There might be cells that inherit endosperm characters in the callus. The PRC2 subunits that are not upregulated in early SE, such as *PaEMF2* and *PaKMT6A2*, are upregulated in desiccated SE (Fig. 6M). *PaEMF2* and *PaKMT6A2*, together with *PaMSI1a* and *PaFIE* may be involved in embryo to seedling transition rather than embryogenesis (Fig. 8).

In Arabidopsis, the *clf swn* mutant fails in leaf-to-callus transition [40] but gains embryonic potential in shoots [41]. These results indicate a complicated role of H3K27me3 in cell totipotency and pluripotency. ECs possess totipotency to differentiate into embryos, which contain the completed body plan needed for plant development. It has been generally assumed that more plastic cells have a more ‘open chromatin architecture’ [42]. Nakamura et al. (2020) have reported a lower H3K27me3 level in EC than in NEC. Their EC and NEC were induced from ZEs and seedling hypocotyls, respectively. They reported a similar H3K27me3 profiles between NEC and seedling hypocotyls. The H3K27me3 profiles are speculated to be at least partly inherited from the explants [30]. It is known that with the plant maturation the induction of EC become more and more difficult. Failure in H3K27me3 demethylation might be related with the loss of somatic embryogenesis competency in the matured tissues.

In our study, the EC and NEC were both induced from ZEs but showed distinct embryonic competency. Four PRC2 subunit genes, *PaKMT6A2*, *PaEMF2, PaMSI1a* and *PaMSI1b*, were more highly expressed in NEC (Fig. 6M). It worth to note that three of these four genes were proposed to be involved in embryo to seedling transition (Fig. 8). *PaKMT6A2* is the enzymatic catalytic subunit of PRC2. While *PaEMF2* is rate-limiting for the enzymatic activity of PRC2 [43]. In Arabidopsis, AtMSI1 is present not only in all three PRC2 complexes but is also a fundamental member of the CAF-1 complex [44]. One of the many functions of CAF-1 is the transmission of H3K27me3 in plant cells through cell division [45, 46]. High expression of these genes may inhibit the H3K27me3 demethylation of NECs either by enhancing PRC2 activity or maintaining H3K27me3 markers during cell proliferation (Fig. 8). On the other hand, it has been reported that DNA methylation, which is critical for embryonic gene expression in both angiosperms and gymnosperms [47, 48], decreases the binding of an H3K27me3 demethylase, REF6, to target sequences [49]. Further studies are necessary to investigate whether the demethylation process is dominated by H3K27me3 demethylases, the inhibition of H3K27me3 transmission or both.

We observed an overlapping of H3K27me3 deposits with meristem regions during embryogenesis (Fig. 7). The number of H3K27me3-marked loci in SAM is more than 1.7 times that of whole seedlings in Arabidopsis [50, 51]. In pluripotent cell-containing tissues, such as roots and shoots, PRC2 depletion induced cell reprogramming with or without hormone application. Embryonic-related transcription factors, such as *ABI3*, *CUCs, ESR2/DRNLs* and *FUS3*, were simultaneously ectopically upregulated in these PRC2-depleted tissues [41, 52]. Homologs of these genes were also found to be released from H3K27me3 markers in the EC but not the NEC in *P. abies* [30]. In contrast, PRC2-depleted leaves can adopt their embryonic identity tissue. Callus formation from leaf explants experiences a tissue identity transition from leaf to root, during which H3K27me3 levels decreased first at certain auxin-pathway genes and then increased at specific leaf genes but decreased at a number of root-regulatory genes [40]. It is proposed that H3K27me3 markers are mostly enriched in pluripotent stem cells. To gain embryonic competency, H3K27me3 markers need to be subtly erased from chromatin with specific programs depending on the original tissues. Conversely, to further specify the cell types, specific tissue-regulatory genes need to be released from H3K27me3 repression while the overall H3K27me3 profiles are maintained.

It worth to note that H3K27me3 deposits were observed in the basal part of the embryo (Fig. 7B). In conifers, the embryonal proper and the suspensor are conjectured to be separated by a type of conifer-specific distal stem cell called the embryonal tube cell, which gives rise to apical meristematic daughter cells in the embryonal proper and basal vacuolated suspensor cells [37]. It is difficult to distinguish these cells under a microscope because they are anatomically more or less similar to the rest of the cells in the embryonal proper. Our work may provide evidence for the existence of stem cells in the basal part of the embryonal proper in conifers.

## Conclusion

In this work, we presented the identification and characterization of the *P. abies* PRC2 core component gene *PaFIE*; two *p55/MSI* homologs *PaMSI1a* and *PaMSI1b*; two histone methyltransferase genes *PaKMT6A2* and *PaKMT6A4;* a *Su(z)12* homolog *PaEMF2* and a *PaEMF2-like* fragment. Phylogenetic analysis suggests that different PRC2 subunits of gymnosperms have evolved asynchronously. Differential expression of these PRC2 core component genes and the deposition of H3K27me3 marks in different tissues or embryo developmental stages imply subtle regulation of H3K27me3 reprogramming during embryogenesis. Our work is the first comprehensive study of *P. abies* PRC2 subunits with a special focus on embryo development, which will guide further research on conifer embryonic potential and embryogenesis.

## Materials and methods

### Plant material

The strobilus of *Picea abies* L. Karst was collected at the Xiaolongshan Forestry Centre of Tian Shui, Gansu Province (lat. 33°35′12″, long. 106°13′10″ E) in 2022. The early seeds were collected on 12 June. The late endosperms and the late zygotic embryos (ZE) were isolated from the seeds collected on 4 July. Mature endosperms and mature ZE were isolated from seeds collected on 20 July. The isolations were performed by hand under stereomicroscope. There might be suspensor remains in the endosperms. The callus 21Pa1-3 and 21Pa1-4, which were used in our study, were induced from ZEs collected at the same location on 10–20 July 2020. Line 21Pa1-4 (embryonic callus, EC) shows a high differentiation rate of somatic embryos (SEs). No SE differentiation could be observed in line 21Pa1-3 (nonembryonic callus, NEC).

SEs were cultured as described previously [53]. Briefly, calli from 21Pa1-3 and 21Pa1-4 were maintained on solidified proliferation medium containing the plant growth regulators (PGRs) auxin and cytokinin. Cultures were transferred to prematuration medium lacking PGRs for one week to stimulate the differentiation of early SE. For the development of intermediate SE, late SE, maturing SE and matured SE, the cultures were transferred to maturation medium supplemented with abscisic acid (ABA). After partial desiccation, the desiccated SE were germinated for 4 weeks. Samples were photographed using an ultradepth microscope (Digital Microscope Leica DVM6, Hesse-Darmstadt, Germany).

### RNA isolation and cDNA synthesis

For RNA isolation and protein extraction, samples were frozen in liquid nitrogen, ground into powder and stored at − 80 °C after collection. Total RNA was isolated using the RNAprep Pure Plant Plus kit (TIANGEN, Beijing) according to the manufacturer’s instructions. RNA quality was assessed and checked by RNase-free agarose gel electrophoresis. For each sample, 1 µg of total RNA was reverse transcribed with the PrimeScript™ RT reagent Kit (Takara, Beijing) using an equimolar ratio of random and oligo-dT primers according to the manufacturer’s instructions.

### Cloning of *P. abies* PRC2 core component genes and protein domain analysis

Gene-specific primers of the *P. abies* PRC2 core component genes were designed based on the published and aligned *Picea* sequences from NCBI (http://blast.ncbi.nlm.nih.gov/Blast.cgi) and the *P. abies* genome database (PlantGenIE.org: Home) (Table S1). The CDSs of *PaFIE*, *PaMSI1a* and *PaMSI1b* were cloned into pMD19-T (Takara, Beijing) and sequenced. The VEFS-BOX of *PaEMF2* and the SET domain of *PaKMT6As* were identified by PCR using degenerate primers. PCR fragments of expected size (approx. 400 and 350 bp, respectively) were cloned and sequenced. Their full-length coding sequences (CDSs) were obtained by 5’/3’ RACE technology (GeneRacer kit, Invitrogen). All cDNAs were cloned from calli. The full-length CDSs were cloned and ligated into pMD19-T (Takara, Beijing). The accessions are listed in Table S2. Their domain organization was analyzed using the HMMER website of Search Pfam [54]. Domains were also verified and named according to the SMART database (http://smart.embl-heidelberg.de/). DOG2.0 was used to generate the pictures [55].

### Phylogenetic analysis

The amino acid sequences of the PRC2 core component genes of *Zea mays* (*Zm*), *Volvox carteri* (*Vc*), *Vitis vinifera* (*Vv*), *Selaginella moellendorffii* (*Sm*), *Populus trichocarpa* (*Pt*), *Ostreococcus lucimarinus* (*Ol*), *Oryza sativa* (*Os*), *Nymphaea colorata* (*Nc*), *Citrus sinensis* (*Cs*), *Chlamydomonas reinhardtii* (*Cr*), *Ceratopteris richardii* (*Cer*), *Brachypodium distachyon* (*Bd*), *Arabidopsis thaliana* (*At*) and *Physcomitrium patens* (*Pp*) were obtained from Phytozome 13 [56]. The nucleotide sequences of *Pseudotsuga menziesii, Taxus baccata*, *Gnetum montanum* and *Pinus sylvestris* were obtained from PLAZA gymnosperm (v1.0). The nucleotide sequences of *Ginkgo biloba (Gb)* were obtained from the Ginkgo Database-Genome (ginkgo.zju.edu.cn) [34, 57] (Supplementary File 2). Only genes containing at least one conserved domain were included in the analysis. Sequences were aligned using the MUSCLE plug-in in MEGA-X [58] and edited manually. Phylogenetic analyses were performed using maximum likelihood with IQ-TREE2 [59]. The best models were selected automatically by the plug-in in IQ-TREE2. Support was calculated using the rapid bootstrap method with 1000 bootstrap replicates. *Drosophila melanogaster* (*Dm*) sequences were used as outgroups. The trees were modified using Interactive Tree Of Life (iTOL) v5 [60].

### Quantitative real-time PCR

Quantitative real-time PCR (qRT‒PCR) was performed on LightCycler 480 using KAPA SYBR LightCycler 480 and 384-well PCR plates with adhesive seals (Roche, USA). Transcript levels were calculated using the **2^-**ΔΔCt method. Two reference genes [37], *CELL DIVISION CONTROL2* (*PaCDC2*) and *ELONGATION FACTOR 1* (*PaEF1*), were used. Two or three biological replicates, each with three technical replicates, were performed for each test. All data were relative to the transcript level of *PaMSI1a* in NEC or in early seed. Shapiro-Wilk was used to check normal distribution of the samples. The samples were normally distributed. Data were analyzed by ANOVA followed by post-hoc test. The primer sequences are listed in Table S3.

### H3K27me3 immunolocalization

The following materials were used for H3K27me3 immunolocalization: seeds collected on 4 July and 20 July 2022 and calli from 21Pa1-3 and 21Pa1-4. The samples were fixed in paraformaldehyde at + 4 °C overnight and embedded in paraffin (Leica Biosystems, Shanghai, China) according to A Karlgren, J Carlsson, N Gyllenstrand, U Lagercrantz and JF Sundstrom [61]. The wax blocks were stored at + 4 °C before using. Section (10 μm) were prepared using Lecia RM2245 (Lecia, Germany). The slides were incubated at + 42 °C overnight. Deparaffinize and dehydrate was performed by washing 3 × 10 min in dimethylbenzene and in serial diluted EtOH. Sections were immersed into 3% H_2_O_2_ for 5 min at room temperature and washed three time with ddH_2_O before blocked with 5% (w⁄v) BSA at 37 °C for 1 h. The primary antibody raised against H3K27me3 (rabbit; A2363; Abclonal, China)was diluted in 1% (w⁄v) BSA at a 1:100 concentration and dropped onto the samples. The sections were incubated for 2 h at 37 °C. Omission of the primary antibody was realized as a control. Immunostaining was performed using a soluble labeled streptavidin biotin (LSAB)/horseradish peroxidase (HRP) complex kit (Solarbio, Beijing, China). Sections were washed three times with PBS for 5 min and then incubated in secondary antibody (sheep anti-rabbit IgG, Solarbio, Beijing, China) for 1 h at room temperature in the dark. The secondary antibody was used at a 1:40 dilution in 1% BSA in PBS buffer. Then sections were washed 3 × 5 min in PBS. The chromogen substrate [3,3’-diaminobenzidine (DAB)] was added in a dark room, and once the brown color appeared, the slides were submerged in water to stop the reaction and then stained with hematoxylin for 30 s. The sections were washed again before viewing using an OLYMPUS BX51 (OLYMPUS, Japan).

## Electronic supplementary material

Below is the link to the electronic supplementary material.

Supplementary Material 1

Supplementary Material 2

Supplementary Material 3

## Data Availability

The datasets and plant materials used and/or analyzed during the current study available from the corresponding author on reasonable request.

## Abbreviations

PRC2: Polycomb repressive complex 2
EC: Embryonic callus
NEC: Nonembryonic callus
ZE: Zygotic embryo
SE: Somatic embryo
