# Supplementary material for: Analysis of polycomb repressive complex 2 (PRC2) subunits in Picea abies with a focus on embryo development
Source: BMC Plant Biol. 2023 Jul 1;23:347. doi: 10.1186/s12870-023-04359-9 (PMC10314529; doi:10.1186/s12870-023-04359-9)
Supplement: Supplementary file 3 — Supplementary Material 3 [file 12870_2023_4359_MOESM3_ESM.doc]

**Supplements**

**Table S1** Blast the PRC2 subunit homologs in *Picea* species

| Arabidopsis Genes | Arabidopsis Accession | gene ID of  *Picea* homologs | Sequence length | Species |
| --- | --- | --- | --- | --- |
| *FIE* | AT3G20740 | MA_33589g0010 | 619bp | *P. abies* |
|  |  | MA_18348g0010 | 375 bp | *P. abies* |
|  |  | EF085760.1 | 1920 bp | *P. sitchensis* |
| *MSI1* | AT5G58230 | MA_142023g0010 | 381 bp | *P. abies* |
|  |  | MA_70799g0010 | 582 bp | *P. abies* |
|  |  | EF678731.1 | 1773bp | *P. sitchensis* |
|  |  | BT123192.1 | 1437bp | *P. sitchensis* |
| *EMF2, FIS2* and *VRN2* | AT5G51230  AT2G35670  AT4G16845 | MA_10432142g0010 | 1116 bp | *P. abies* |
|  |  | MA_10436590g0020 | 765bp | *P. abies* |
|  |  | BT110324.1 | 1245 bp | *P. glauca* |
|  |  | BT122256.1 | 787bp | *P. sitchensis* |
| *CLF, MEA* and *SWN* | AT2G23380 | MA_7025g0010 | 447 bp | *P. abies* |
|  | AT4G02020 | MA_10430132g0010 | 998 bp | *P. abies* |
|  | AT1G02580 |  |  |  |

**Table S2** Accessions of the *Picea abies PRC2* subunit CDS.

| *P. abies* homologs | Length of CDS | Length of protein | Genbank Accession |
| --- | --- | --- | --- |
| PaFIE | 1110 bp | 369 aa | OQ605891 |
| PaMSI1a | 1266 bp | 421 aa | OQ605889 |
| PaMSI1b | 1206 bp | 401 aa | OQ605890 |
| PaEMF2 | 1746 bp | 581 aa | OQ605894 |
| PaEMF2-like fragment | 651 bp | 130 aa | OQ605895 |
| PaKMT6A2 | 2850 bp | 949 aa | OQ605892 |
| PaKMT6A4 | 2820 bp | 939 aa | OQ605893 |

**Table S3** Primer list for qRT-PCR

| **qPCR Primer list** | **Forward** | **Reverse** |
| --- | --- | --- |
| PaEF1 | CACCTTGGGAGTGAAGCAAATG | GGGAGTAGTGGCATCCATCTTG |
| PaCDC2 | TCCGACGGGTGCAGAGAA | GCTCCATTC AGCCTGATTCAA |
| PaFIE | TTGGGTACAGATGCGGCGATAG | TGGACCTTTTCATTTGCGACAT |
| PaEMF2 | CACATATCTTGGGCATGCGA | AAGCCCTTGAAAACAGCATC |
| PaEMF2-like fragment | CGGATGGCCATATCCCTTGG | AGGGCGTGATGAGTTACTGG |
| PaKMT6A2 | AGTCTGATGTTGCTGGATGG | GCTCCTCACCAGATGCAATG |
| PaKMT6A4 | CGAGAAGCTGATAAGCGAGG | GCCCTATCTGGCTCATAACG |
| PaMSI1a | GAGGGGTCATAATACAGAAGGTT | CTTGCGCAGATCAAAAAGTT |
| PaMSI1b | ACATGCGTACAGCTGAACCTAACA | GTGCCCTCCATGAACGAAAAGTAA |


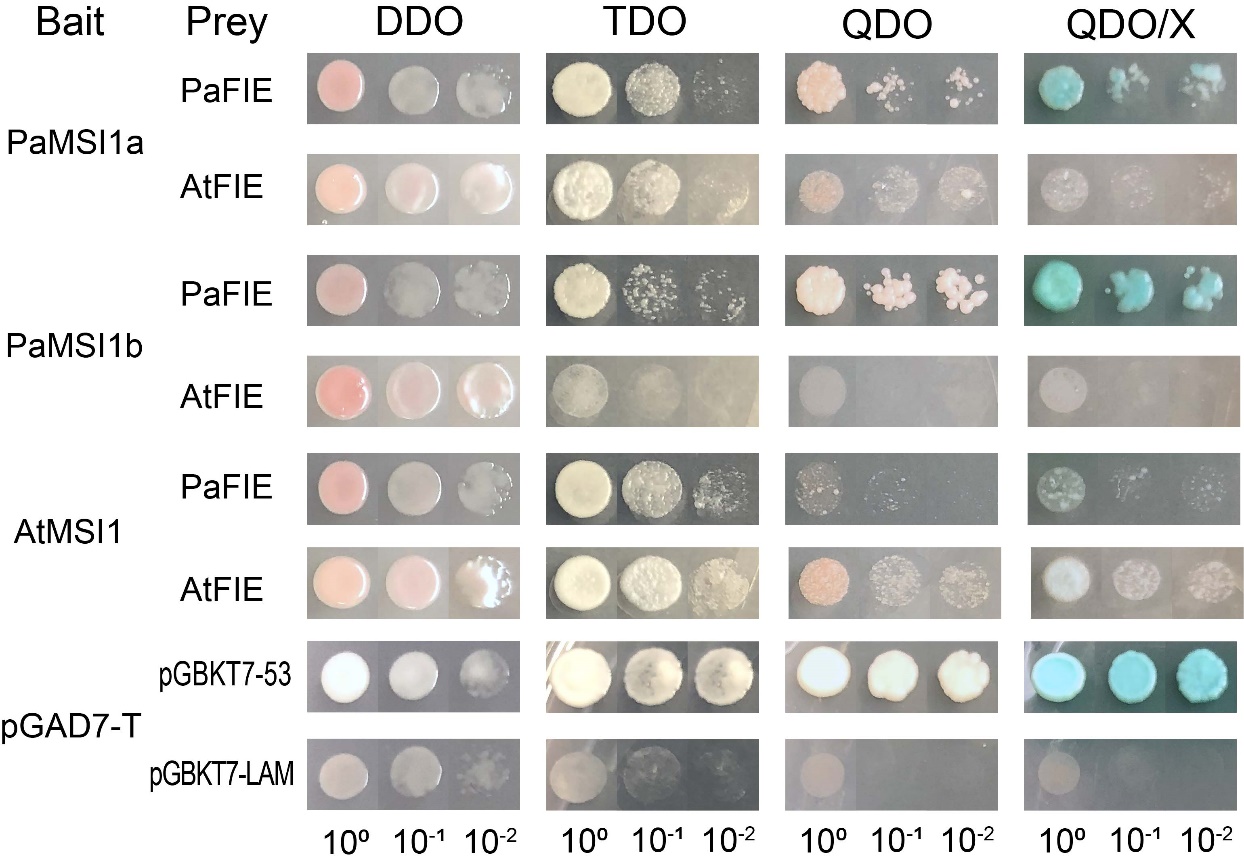


Figure S1 Yeast-two-Hybrid assay showed that both PaMSI1a and PaMSI1b could interact with PaFIE. pGADT7-T cotransformed with pGBKT7-53 and pGBKT7-LAM were used as positive and negative controls, respectively. Mediums: DDO, double dropout supplements; TDO, triple dropout supplements; QDO, quadruple dropout supplements and QDO/X, QDO plus X-a-gal. The interactions can be judged by the growth of yeast on selective medium (TDO, QDO and QDO/X). AD-PaFIE co-transformed with BD-PaMSI1a or BD-PaMSI1b and AD-AtFIE co-transformed with BD-AtMSI1 grew on all the selective medium. AD-AtFIE co-transformed with BD-PaMSI1a and AD-PaFIE co-transformed with BD-AtMSI1 grew on the weak selective medium TDO. AD-AtFIE co-transformed with BD-PaMSI1b did not grow on all the selective medium.

_
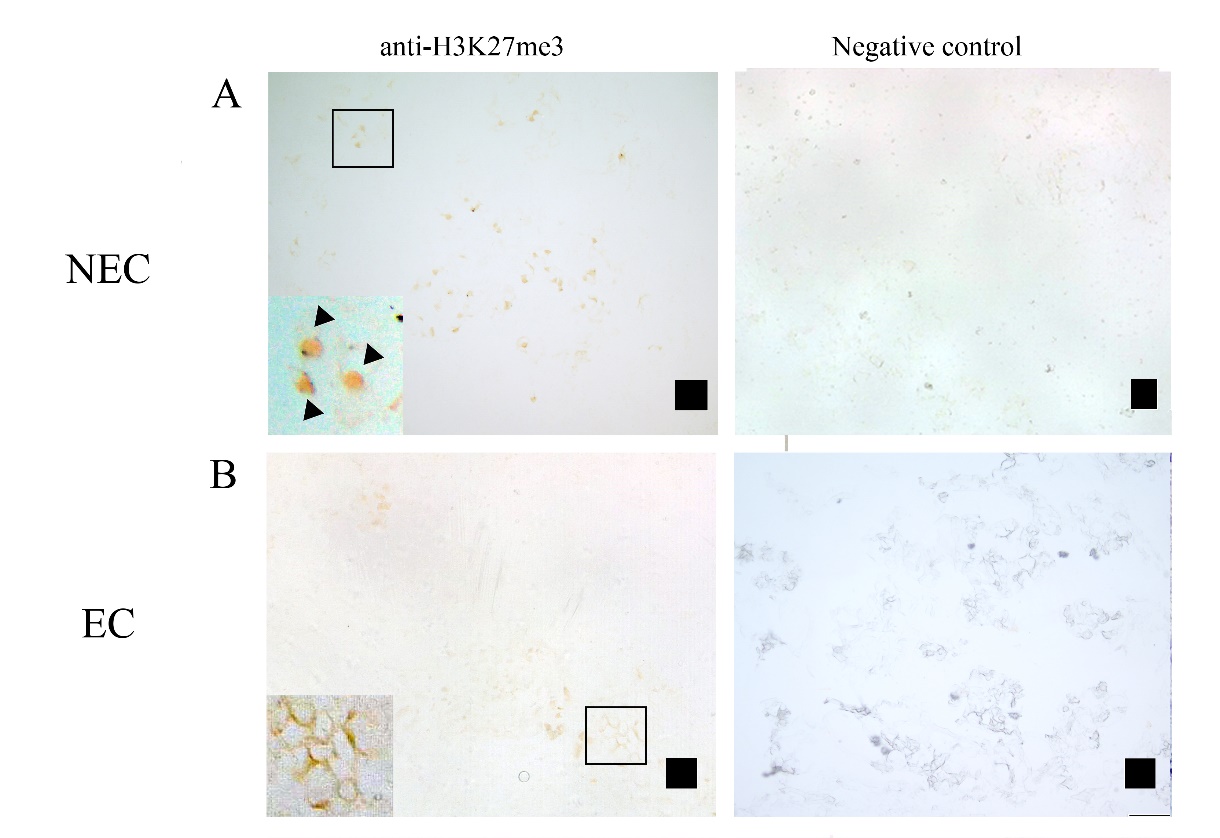
_

Figure S2 H3K27me3 immunohistochemistry of (A) non-embryonic callus (NEC) and (B) embryonic callus (EC) of *Picea abies*. Bar 100 μm. The left column was the anti-H3K27me3 group, while the right column was the corresponding negative control. The area in the black square were showed with higher magnification on the bottom left. The triangles in A-left point the H3K27me3 deposits detected in NEC. Few H3K27me3 deposit could be detected in EC.
